# Genomic and statistical models to characterize Streptococcus pneumoniae transmission patterns in Malawi

**DOI:** 10.1099/mgen.0.001667

**Published:** 2026-04-09

**Authors:** Rory Cave, James Chirombo, Uri Obolski, Sophie Belman, Akuzike Kalizang'oma, Thandie S. Mwalukomo, Arox Kamng’ona, Comfort Brown, Jacquline Msefula, Farouck Bonomali, Roseline Nyirenda, Todd D. Swarthout, Brenda Kwambana-Adams, Neil French, Robert S. Heyderman

**Affiliations:** 1 NIHR Global Health Research Group on Vaccines to Control Respiratory Pathogens and AMR across Africa, Institute of Infection, Immunity and Transplantation, University College London, London, UK; 2 Malawi Liverpool Wellcome Programme, Blantyre, Malawi; 3 Department Clinical Infection, Microbiology and Immunology, Institute of Infection Veterinary & Ecological Science, University of Liverpool, Liverpool, UK; 4 Department of Epidemiology and Preventive Medicine, School of Public Health, Faculty of Medicine, Tel Aviv University, Tel Aviv, Israel; 5 Global Health Resilience Group, Earth Sciences Department, Barcelona Supercomputing Center - Centro Nacional de Supercomputación, Barcelona, Spain; 6 Kamuzu University of Health Sciences, Blantyre, Malawi; 7 Julius Center for Health Sciences and Primary Care, University Medical Centre Utrecht, Utrecht, Netherlands

**Keywords:** antimicrobial resistance, Malawi, phylogenetic, *Streptococcus pneumoniae*, transmission

## Abstract

Controlling the carriage and transmission of *Streptococcus pneumoniae* in children from high-burden settings is critical for disease prevention. To investigate the rate and drivers of transmission following pneumococcal conjugate vaccine (PCV) introduction, we estimated evolutionary divergence times using whole-genome sequences of *S. pneumoniae* from 1,617 children in Blantyre, Malawi (2015–2019). The cohort included PCV13-vaccinated children aged 2–7 years and unvaccinated children aged 5–10 years who were vaccine ineligible at the time of rollout into routine use. Using generalized additive mixed models and relative risk frameworks that incorporated household geospatial distances, we found that pneumococcal lineages spread widely across Blantyre within ~4 years, with transmission most likely between neighbouring households. Logistic regression and random forest models identified higher transmission risk among preschool-aged children in densely populated, higher socioeconomic areas. Recent transmission events were primarily associated with expanding, non-vaccine serotype lineages that were non-susceptible to penicillin. These findings highlight the potential for extended valency PCVs to reduce the spread of disease-causing and antimicrobial-resistant pneumococcal lineages among preschool-aged children, particularly in high-density areas – thereby strengthening herd protection for vulnerable groups such as young infants and individuals living with HIV.

**Impact Statement**

*Streptococcus pneumoniae* remains a leading bacterial cause of pneumonia, meningitis and sepsis in children worldwide. Although pneumococcal conjugate vaccines are widely implemented across many low- and middle-income countries, control of pneumococcal disease remains incomplete. Vaccine-serotype carriage and disease persist, and antimicrobial-resistant lineages continue to emerge. Understanding transmission dynamics is crucial to guide targeted interventions that enhance vaccine effectiveness and reduce the global burden of pneumococcal disease.

## Data Summary

R and Python code used to plot the generalized additive mixed model and random forest model can be found in the Git repository: https://github.com/rorycave/Blantyre_SPN_geospace_paper. Whole-genome sequence data are available from BioProject PRJNA1011974. A list of strain accession numbers can be found in Table S1, available with the online version of this article.

## Introduction

Understanding transmission dynamics is essential for designing targeted interventions to control *Streptococcus pneumoniae* spread in high-burden settings. While compartmental and phylogenetic models have advanced our knowledge of pathogens – such as SARS-CoV-2 and *Mycobacterium tuberculosis* – bacterial species like *S. pneumoniae* present unique challenges due to high carriage prevalence, extensive strain diversity and frequent co-colonization [1,4].

*S. pneumoniae* is a respiratory pathogen responsible for a substantial global burden of pneumonia, meningitis and sepsis, accounting for ~300,000 deaths annually in children under five [5,6]. Nasopharyngeal (NP) carriage is typically asymptomatic yet is a pre-requisite for both transmission and disease [7]. Spread occurs via direct person-to-person contact through respiratory droplets, particularly among children in crowded environments [8,10]. Over 100 serotypes and 900 lineages – defined by Global Pneumococcal Sequence Type (GPSC) – co-circulate, often coexisting in the nasopharynx, particularly in resource-limited settings [11].

Pneumococcal conjugate vaccines (PCVs) have been incorporated into routine immunization programmes in more than 160 countries, reducing carriage, transmission and disease [7,12]. However, despite strong direct protection, herd immunity has remained suboptimal in many settings. Mathematical models combined with pneumococcal genomic data have estimated inter-country spread rates, mother-to-child transmission and the impact of vaccination on invasive pneumococcal disease (IPD) incidence across age groups and geographies [13,17]. Although factors influencing carriage have been extensively studied, less is known about how epidemiological and bacterial factors influence *S. pneumoniae* spread in densely populated urban areas following PCV introduction.

In Malawi, following routine PCV13 introduction in 2011, herd protection against IPD – particularly among unvaccinated children and adults living with HIV (PLHIV) – has been limited, despite vaccine coverage exceeding 90% [18,21]. This may reflect persistent vaccine-serotype carriage and age-related factors and a sustained high force of infection [20,22]. Moreover, shifts in population structure have led to genotypes with virulence and antimicrobial resistance (AMR) profiles that confer competitive advantage, as well as capsule-locus switch variant lineages – particularly genotypes associated with serotypes 3, 14 and 23F – that facilitate vaccine escape [23,24]. We hypothesize that vaccine-escape lineages emerge and persist through short-range transmission among young children, amplified by high antibiotic exposure driving AMR selection [25].

To test this hypothesis, we integrated large-scale longitudinal genomic and epidemiological data from a high-burden urban population in Malawi. By combining time-calibrated phylogenetics, spatial generalized additive mixed models (GAMMs) and machine-learning approaches, an application not previously employed on densely sampled carriage data in an urban African setting, we leveraged large long-standing carriage surveys to quantify the spatial–temporal spread of pneumococcal lineages and identify the human and bacterial factors driving local transmission.

## Methods

### Setting and study population

Blantyre, a city in southern Malawi (228 km²), has an urban population of ~1 million, with a growth rate of 3.9% and an average population density of 3,006 people/km². Within Blantyre, certain high-density areas reach up to 34,602 people/km². Participants were recruited in Blantyre between 2015 and 2019 as part of the Pneumococcal Carriage in Vulnerable Populations in Africa (PCVPA) study, following the introduction of PCV13 into the routine immunization schedule in November 2011. The PCVPA study methods have been detailed previously [20].

Briefly, participants included healthy infants aged 4–8 weeks prior to their first PCV13 dose, healthy children aged 18 weeks to 7 years who had received PCV13 via routine immunization or catch-up campaigns and healthy children aged 3–10 years who were age-ineligible for PCV13 (born on or before 11 November 2010). Epidemiological data collected included GPS coordinates of the household, household composition, NP swab collection date, participant age and gender, vaccination status and socioeconomic status. Annual population density data for Blantyre were sourced from the WorldPop research programme [26] which generates modelled outputs using census, geographic and settlement data to estimate the population within 100 m×100 m grid cells, with confidence intervals (CIs).

### Isolates and whole-genome sequences

*S. pneumoniae* was isolated from NP samples by culture, and DNA was extracted from individual colonies as previously described [27]. Draft genome assemblies were obtained from isolates collected between 2015 and 2019 from PCV-vaccinated children aged 2–7 years (*n*=1,882) and PCV-unvaccinated children aged 5–10 years (*n*=600) [20].

### Genetic typing and antibiotic resistance testing

Whole-genome sequences were analysed using Pathogenwatch [28] to determine serotypes, genetic lineages and AMR. Lineages were defined using Global Pneumococcal Sequence Cluster (GPSC) via POPpunk v2.7.0 [29] and MLST. Penicillin MICs were predicted *in silico* using the SPN-PBP-AMR algorithm with EUCAST 2024 breakpoints [30,32]. Other AMR genes and mutations were also identified through Pathogenwatch.

### Bayesian time-calibrated phylogenetic tree construction

For each GPSC, a time-calibrated Bayesian phylogenetic tree was constructed to extract divergence time. ReferenceSeeker was used to identify the closest complete genome from GenBank [33,34], isolates were aligned using Parsnp v1.0 [35] and detectable recombinant fragments were identified and removed with Gubbins v3.3.1 [36]. Phylogenies were constructed using BactDating v1.1.2 with the mixedcarc model [37]. Trees with all parameters converged (ESS>100) were retained. Pairwise divergence times were extracted using the rrspread R package (https://github.com/hsuehchien66/rrspread_v2).

### Statistical analysis of pairwise distance and divergence from the phylogenetic tree

A GAMM with a Gaussian distribution was fitted using the mgcv v1.9–1 R package to explore the relationship between spatial and genetic divergence:

The model formula was specified as follows:


$$Y_{ij}=ƒ\left(T_{ij}\right)+b_{g[i]}+b_{s[i]}+\varepsilon_{ij}$$

Where:

$Y_{ij}$ : geographic distance (km) between isolates i and j

$T_{ij}$: estimated divergence time (years) from the Bayesian phylogeny

$ƒ\left(T_{ij}\right):$ thin-plate spline smooth

$b_{g[i]}$ and $b_{s[i]}$: random intercept to account for non-independence due to sample-level effects

$\varepsilon_{ij}∼N\left(0,\sigma^{2}\right)$ is the residual error

The saturation point of the GAMM curve was defined as the first instance where the derivative of the curve fell below 0.1, indicating a plateau in the relationship between divergence time and geographic distance.

### Relative risk calculation framework

We estimated the relative risk (RR) of recent transmission events by comparing the proportion of isolate pairs with low divergence (<1 year) within specific spatial or age intervals to those outside those intervals. Divergence time and distance were binned into the following intervals:

Divergence time (years): 0–1, 1–2, 2–3, 3–4 and 4–5

Geographic distance (km): 0–0.075, 0.075–0.5, 0.5–1, 1–2, 2–3, 3–4 and 4–5

The RR was calculated using a formula adapted from [16]:


$$RR_{i,d}=\;\frac{n_{i,d}/N_{i}}{\left(N_{\neg i,d}\right)/N_{\neg i,d}}$$

Where:

$n_{i,d}$: number of isolate pairs within distance interval i and divergence group d

$N_{i}$: total number of isolate pairs within distance interval i

$N_{\neg i,d}$: number of isolate pairs outside the distance interval i but within the divergence group d

$N_{\neg i,d}$: total number of isolate pairs outside the distance interval i

RR was also calculated based on the likelihood of transmission between children as a function of their age difference, grouped into the following intervals: 0–1, 1–2, 2–3, 3–4, 4–5 and 5–6 years. The formula used was as follows:


$$RR_{j,a}=\frac{m_{j,a}/m_{j}}{(m_{\neg j,a})/m_{\neg j}}$$

Where:

$m_{j,a}$: number of isolate pairs with divergence <1 year within the age difference interval j

$m_{j}$: total number of isolate pairs within the age difference interval j

$m_{\neg j,a}$: number of isolate pairs with divergence <1 year outside the age difference interval j

*m*¬*j*: total number of isolate pairs outside the age difference interval j

CIs for RR were estimated using bootstrap resampling (*n*=20). For each bootstrap iteration, individuals were resampled with replacement, and RR was recalculated.

### Random forest and generalized linear mixed-effects models

A two-stage analytical approach was employed to identify bacterial and human factors most strongly associated with pneumococcal transmission. In the first stage, a random forest classifier was trained to predict transmission events, defined as isolate pairs with genetic divergence below the saturation threshold for both time and distance [38,40]. The dataset was randomly split into a training set comprising 80% of the data and an independent test set comprising the remaining 20%, using stratification to preserve the class balance between recently transmitted and non-transmitted isolate pairs.

Candidate predictors included child age, survey year, socioeconomic score, household composition, GPSC, serotype group (PCV13 vs non-PCV13), AMR genotype and penicillin minimum inhibitory concentration (MIC). All feature selection, hyperparameter optimization and cross-validation were performed exclusively on the training dataset, with final model performance evaluated on the held-out test dataset. Categorical variables were one-hot encoded. Hyperparameter optimization was conducted using Optuna (v4.0), and feature selection was performed using recursive feature elimination with cross-validation. Model performance was evaluated using receiver operating characteristic- area under curve (ROC-AUC), precision-recall- area under curve (PR-AUC) and standard classification metrics. Feature importance was interpreted using Shapley Additive exPlanations (SHAP) (v0.46). The model was implemented in Python using scikit-learn (v1.5.2).

In the second stage, top-ranked predictors from the machine learning model were evaluated using generalized linear mixed-effects models (GLMMs) in R. The binary outcome captured whether isolate pairs showed evidence of recent transmission. All models were fit using the lme4 package (v1.1-35.4), with GPSC included as a random effect to account for lineage structure. Continuous variables were standardized (z-scored), and univariable GLMMs were fit for each predictor using a binomial error structure and the bobyqa optimizer. Predictors with *P*-values <0.10 were selected for inclusion in a global multivariable GLMM. Model selection was performed using the dredge function from the MuMIn (v1.48.11) package, ranking candidate models by corrected Akaike information criterion. The best-fitting model was retained for interpretation, and odds ratios (ORs), 95% CIs and *P*-values were extracted using the broom.mixed (v0.2.9.6) package.

### Lineage effect analysis

Lineage effects were analysed using a linear mixed model in pyseer v1.13.10, based on the method by Earle *et al*. [41,42]. Genetic distances were calculated with Mash v2.0. Lineages were assigned by GPSC and MLST [43]. Bonferroni correction was adjusted for multiple testing. Changes in lineage prevalence were assessed using chi-squared tests for trend (rstatix v0.7.2).

### Population expansion

Clonal expansion and effective population size were estimated using the Bayesian phylogeny and CaveDive v0.1.1 R package, applying priors from Helekal *et al*. [44].

## Results

**Fig. 1.**
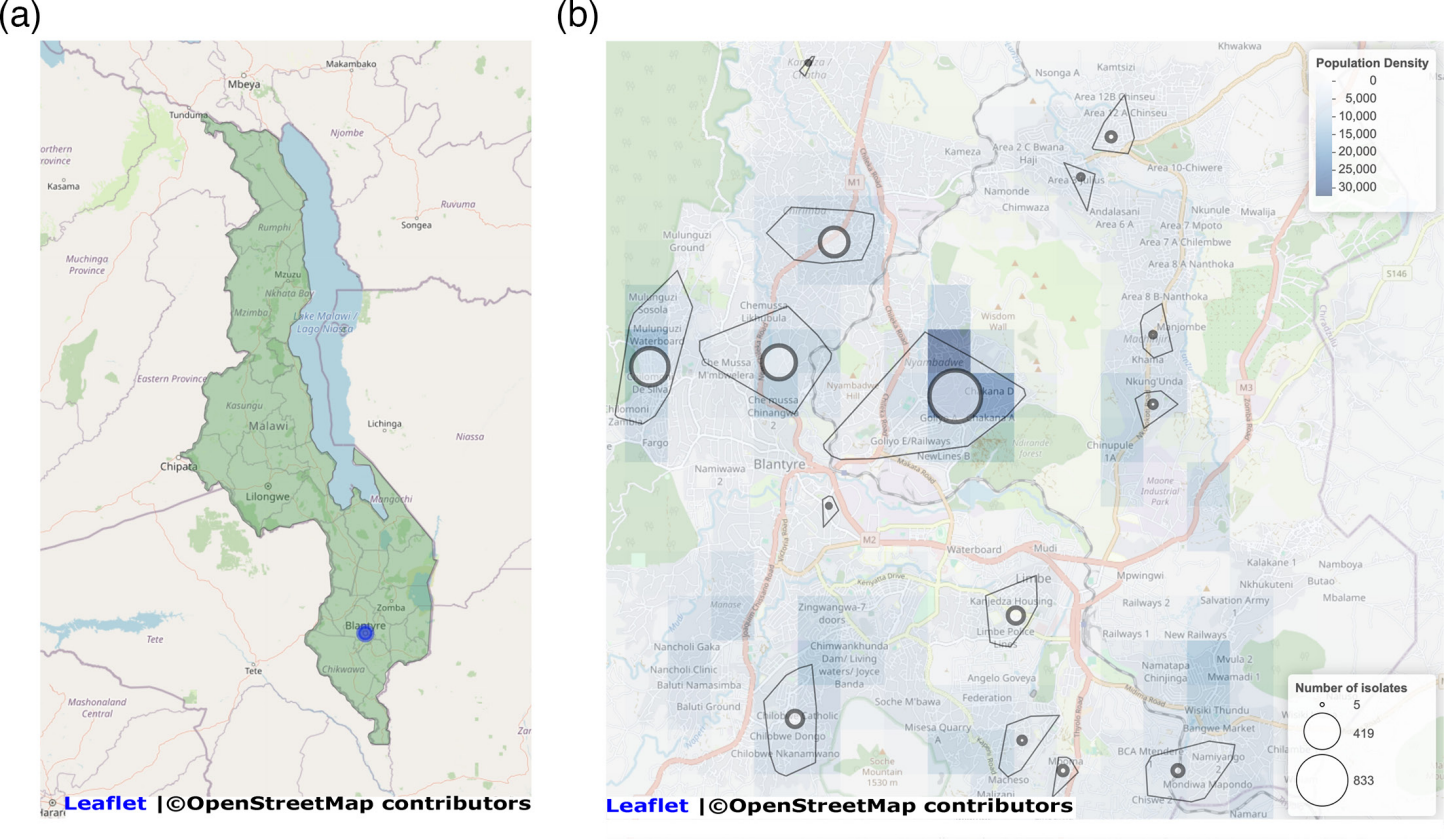
Geographic representation of Malawi and Blantyre. (a) Map of Malawi indicating the location of Blantyre (blue dot). (b) Detailed map of Blantyre illustrating population density, with areas where samples were collected outlined by black convex hulls, and the number of isolates collected from each area represented by marker size. Base map data ©OpenStreetMap contributors and distributed under the Open Database License (ODbL).

We analysed 2,283 carriage isolates collected from children in urban Blantyre between 2015 and 2019 (Fig. 1, Table S1, available in the online Supplementary Material) [23]. Each child contributed one isolate, yielding 59 serotypes – 23.1% (*n*=528) of which were PCV13 vaccine types (VTs). One hundred eighteen GPSC lineages were represented. Based on a genomic analysis, 37.4% of isolates (*n*=854) were penicillin non-susceptible (MIC >0.12 µg ml^−1^, meningitis breakpoint), 30.3% (*n*=692) were tetracycline-resistant and 16% (*n*=366) were erythromycin-resistant.

### Phylogenetic and geographic analyses of pneumococcal spread across Blantyre, Malawi

To infer pairwise divergence times, we used BactDating on recombination-filtered alignments for each GPSC. Of 118 lineages, 31 had sufficient temporal signal to build Bayesian time-calibrated phylogenetic trees, encompassing 1,617 of the 2,283 isolates (Fig. S1). These 31 lineages showed no obvious geographic clustering (Table S1).

**Fig. 2.**
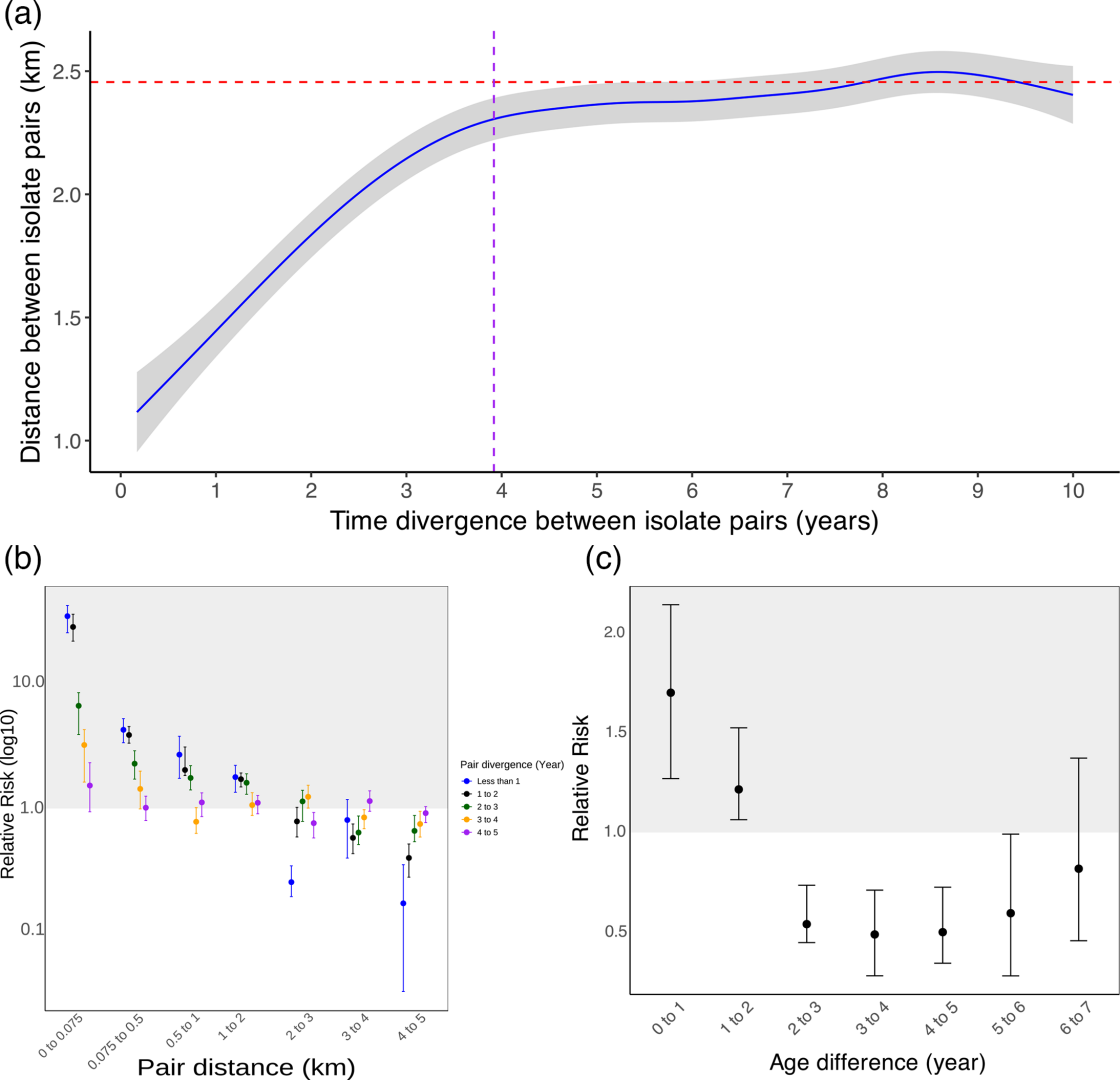
GAMM model and RR analysis. (**a**) A GAM mixed model showing the divergent time between pairs against the distance between pairs. Blue line is the plotted GAM model, grey area is the 95% CI, purple dashed line is the saturation point of the curve and red dashed line is the mean distance of all pairs that have less than 10 years of divergence. (**b**) RR of pneumococcal transmission across spatial distances (0–5 km) and divergence time intervals (0–5 years). RR >1 (red) indicates higher transmission likelihood within the interval. (***c***) RR of transmission between children grouped by age differences (0–6 years). Error bars represent 95% CIs.

Next, we developed a GAMM relating phylogenetic divergence time to the geographic distance between isolate pairs (Fig. 2a). The GAMM explained 76.6% of variance and confirmed that more closely related isolates tend to be found nearer to each other (*P*<0.001). Divergence distance increased with geographic separation but plateaued at ~3.92 years, at which point pairs were on average 2.31 km apart (the mean distance among all pairs diverged by ≤10 years was 2.46 km, range 0–14.3 km). In other words, even in a densely populated, high-carriage setting, pneumococcal lineages spread gradually – taking nearly 4 years to mix broadly across the Blantyre community. Furthermore, recent divergence between isolates sampled nearby in space and time supports the occurrence of ongoing local transmission within the community.

### Estimates of the risk of recent transmission across Blantyre, Malawi

We then calculated the RR of recent transmission – defined as the likelihood that a pair of isolates with a given level of genetic divergence would be found within a specific geographic distance and age difference. RR falls as spatial separation grows: the highest RR occurred for pairs within 75 m (RR 1.53, 95% CI 0.92–2.34) (Fig. 2b). Similarly, RR declined with increasing divergence time, indicating that recent transmission events were more localized. Age differences also mattered: pairs of children aged within 1 year of each other had the highest RR (1.70; 95% CI 1.27–2.20), which dropped for age gaps of 1–2 years (RR 1.22; 95% CI 1.04–1.42) and 2–3 years (RR 0.54; 95% CI 0.35–0.84) (Fig. 2c). Thus, transmission is most likely among similarly aged children living near each other.

### Non-linear effects on transmission probability

**Fig. 3.**
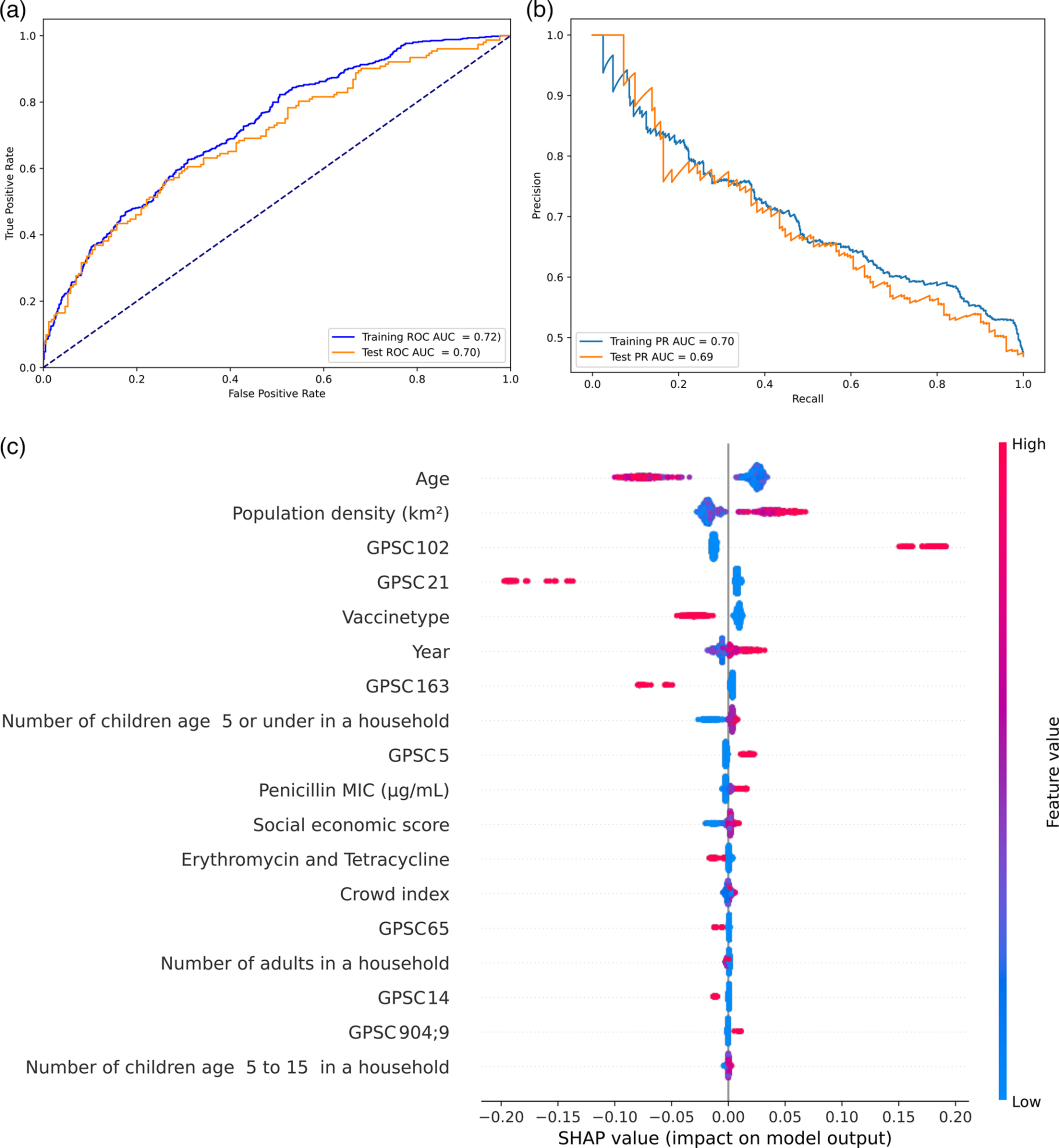
Random forest model fit to predict human and bacterial factors associated with transmission. (**a**) The ROC curve showing the performance of the random forest model against the training and test datasets. (**b**) PR curve of the random forest model on the training and test datasets. (**c**) Beeswarm plot of SHAP values for each feature’s impact on the model’s predictions regarding the likelihood of an isolate being part of recent transmission. The features are displayed in descending order of importance from top to bottom, based on the average absolute SHAP value.

Recognizing that transmission dynamics may be non-linear – particularly as vaccine coverage alters the susceptible population – we first employed a random forest model to capture these complex patterns and identify the most influential predictors. The model was trained on 1,293 isolate pairs (80% of the data) and evaluated on an independent test set of 324 isolate pairs (20%). Using isolate pairs classified as ‘recently transmitted’ (defined as genetic divergence <4 years and spatial separation ≤2.5 km), the model achieved an ROC-AUC of 0.70 (95% CI: 0.64–0.76; Fig. 3a) and a PRAUC of 0.69 (95% CI: 0.62–0.76; Fig. 3b) on an independent test set. Sensitivity was 0.69 (95% CI: 0.61–0.76), specificity 0.55 (95% CI: 0.48–0.63) and G-mean 0.62 (95% CI: 0.57–0.67), indicating a reasonable balance between identifying recent transmission events and limiting false positives.

**Fig. 4.**
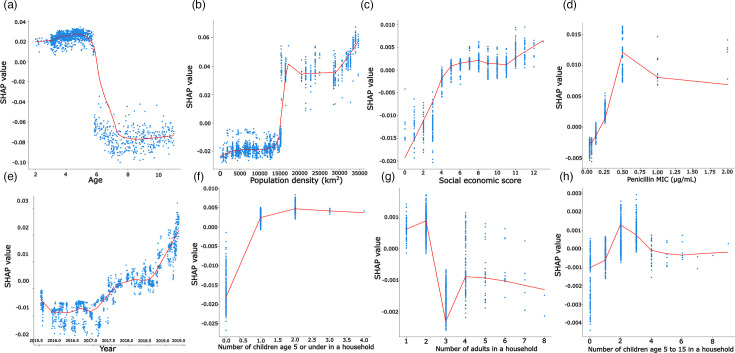
Partial plot of SHAP values for (a) age of child, (**b**) population density, (**c**) socioeconomic score, (**d**) penicillin MIC, (**e**) year of isolation, (**f**) number of children aged 5 or under in a household, (**g**) number of adults in a household and (**h**) number of children aged 5–15 in a household. The red line is the locally estimated scatterplot smoothing trend of the partial plot.

SHAP values identified the top five predictors of recent pneumococcal transmission: child’s age, population density and three bacterial features – lineages GPSC102, GPSC21 and vaccine serotype (Fig. 3c). Although these broadly align with logistic regression findings, the random forest captured non-linear effects more effectively. For instance, children under six exert a much stronger positive influence on transmission probability than older children (Fig. 4a). In Malawi, once children reach 6 years of age, most begin attending school, suggesting that preschool settings drive transmission. Population density exhibited a non-linear relationship: transmission probability rose modestly until ~15,000 people/km², increased sharply between 15,000 and 30,000 people/km², plateaued briefly and then climbed again above 30,000 people/km² (Fig. 4b). This pattern implies that beyond a certain density threshold, additional factors – such as overcrowding, household structure and community infrastructure – further amplify spread. Socioeconomic score likewise showed non-linearity: transmission probabilities level off for scores of 6–11 before rising above 12 (Fig. 4c), indicating that wealthier households may have contact patterns that favour spread in ways not captured by carriage prevalence alone. Penicillin MIC impacted transmission in a non-linear fashion as well: RR increased up to ~0.5 µg ml^−1^ and then declined for higher MICs (Fig. 4d). This suggests that moderate penicillin non-susceptibility strains have enhanced transmission, whereas very high MICs were less frequently observed in carriage during the study period. Limited numbers of isolates with MIC >0.5 µg ml^−1^ likely introduce some noise into these estimates. Erythromycin and tetracycline resistance had minimal impact on transmission probability. Among erythromycin-resistant isolates (*n*=307), 30.29% (*n*=93) had penicillin MICs ≤0.12 µg ml^−1^ (fully susceptible), indicating that erythromycin resistance can emerge independently of penicillin non-susceptibility.

Year of isolation also exhibited a non-linear relationship with transmission: there was a notable rise between 2017 and mid-2018, with another increase after 2019 (Fig. 4e), corresponding to the emergence of penicillin-non-susceptible strains (Wilcoxon *P*<0.001; Fig. S2). Likewise, household composition influenced transmission risk – homes with more children under five or aged 5–15 experienced higher transmission probabilities, whereas those with two or more adults saw reduced spread (Fig. 4f–h). This pattern suggests that multiple young children in a household amplify intra-family transmission, while additional adults may curb child-to-child contact and thus lower overall spread.

### Host and bacterial factors that determine pneumococcal transmission

To assess how human and bacterial factors shape pneumococcal transmission, using predictors identified by the preceding random forest analysis, we fitted a univariable mixed‐effects logistic regression model to inform our multivariable mixed‐effects logistic regression models (Table S2). In line with earlier studies [29,30], transmission odds fell with each additional year of age (adjusted OR 0.82 per year; 95% CI 0.73–0.92; *P*<0.001) but rose in areas of higher population density (adjusted OR 1.31 per km²; 95% CI 1.18–1.46; *P*<0.001) (Table 1).

**Table 1.** Univariate and multivariate mixed effect logistic regression model on human and bacterial characteristics associated with transmission. Transmission was significantly more likely among younger children, in higher density areas and among isolates with higher penicillin MICs

		Univariate			Multivariate	
Characteristic	OR	95% CI	*P*-value	OR	95% CI	*P*-value
Age	0.77	0.70, 0.86	<0.001	0.82	0.73, 0.92	<0.001
Population density (per km²)	1.37	1.23, 1.52	<0.001	1.31	1.18, 1.46	<0.001
Socioeconomic score	1.09	0.99, 1.21	0.092	1.05	1.01, 1.09	0.014
PCV13 VT serotypes	0.59	0.47, 0.75	<0.001	0.54	0.35, 0.83	0.005
Penicillin MIC (μg ml^−1^)	1.24	1.04, 1.47	0.014	1.30	1.09, 1.55	0.004

Pneumococcal vaccine serotypes were significantly less likely to appear in recent transmission events than non-vaccine serotypes (adjusted OR 0.54; 95% CI 0.35–0.83; *P*=0.005), whereas higher penicillin MIC values were positively associated with transmission (adjusted OR 1.30 per μg ml^−1^; 95% CI 1.09–1.55; *P*=0.004). Consistent with this, penicillin MIC increased significantly between isolates collected in July 2017–June 2019 compared to those from July 2015–June 2017 (Wilcoxon *P*<0.001; Figs S2 and S3). These findings support emerging evidence that, in populations with high vaccine coverage and antimicrobial use, vaccine‐escape lineages exhibiting AMR are more likely to spread [17,24, 45].

We observed the expected pattern of higher carriage prevalence among children from lower‐socioeconomic households (adjusted OR 0.77 per socioeconomic score unit; 95% CI 0.90–0.93; *P*<0.001) (Table 2). In contrast, recent transmission was more likely among children from higher‐socioeconomic households (adjusted OR 1.05 per socioeconomic score unit; 95% CI 1.01–1.09; *P*=0.014), suggesting that household wealth and associated behaviours may facilitate pneumococcal spread in ways not reflected by carriage prevalence alone (Table 1). Although the univariate association was not statistically significant (*P*=0.092), the relationship became apparent after adjusting for confounders, indicating that age, population density and serotype distribution may influence the association between socioeconomic status and transmission risk.

**Table 2.** Univariate and multivariate logistic regression model on human factors associated with carriage. Carriage was more prevalent among children from lower socioeconomic households, whereas transmission was more common among children from higher socioeconomic settings

		Univariate			Multivariate	
Characteristic	OR	95% CI	*P*-value	OR	95% CI	*P*-value
Age	0.80	0.76, 0.84	<0.001	0.87	0.82, 0.92	<0.001
Population density (per km²)	1.20	1.14, 1.28	<0.001	1.40	1.08, 1.21	<0.001
Socioeconomic score	0.74	0.70, 0.79	<0.001	0.77	0.73, 0.82	<0.001

### Recent lineage expansion is associated with increased transmission

To verify the lineage effects suggested by the random forest model, we performed a GWAS-style analysis [42]. At the GPSC level, GPSC102 (*P*<0.001), GPSC21 (*P*<0.001) and GPSC163 (*P*=0.01) all showed significant associations with transmission. Of these, only GPSC102 was predicted by the random forest to enhance transmission probability, whereas GPSC21 and GPSC163 appeared to reduce it (Fig. 3c). At the sequence type (ST) and serotype level, GPSC102-ST4423-serotype 23B (*P*=0.001), GPSC5-ST10599-serotype 35B (*P*=0.02), GPSC102-ST10880-serotype 23B (*P*=0.02) and GPSC5-ST10603**-**serotype 15B/C (*P*=0.03) were each significantly linked to community transmission, matching the lineages highlighted by the random forest.

Next, we examined how these GPSCs and STs changed in prevalence over the study period (Fig. S4). Although the overall prevalence of GPSC102 (*P*=0.05), GPSC21 (*P*=0.30), GPSC163 (*P*=0.40) and GPSC5 (*P*=0.80) did not shift significantly, several individual STs did. GPSC102-ST4423 – which first appeared in 2016 – rose by 5.52% (*P*<0.001), and GPSC5-ST10599 increased by 2.68% (*P*<0.01). By contrast, GPSC102-ST10880 declined by 1.86% (*P*=0.002), likely reflecting its penicillin susceptibility, while GPSC5-ST10603 showed no significant change (*P*=0.30). The concurrent rise of the penicillin-resistant ST4423 alongside the fall of ST10880 suggests lineage replacement driven by antibiotic pressure.

**Fig. 5.**
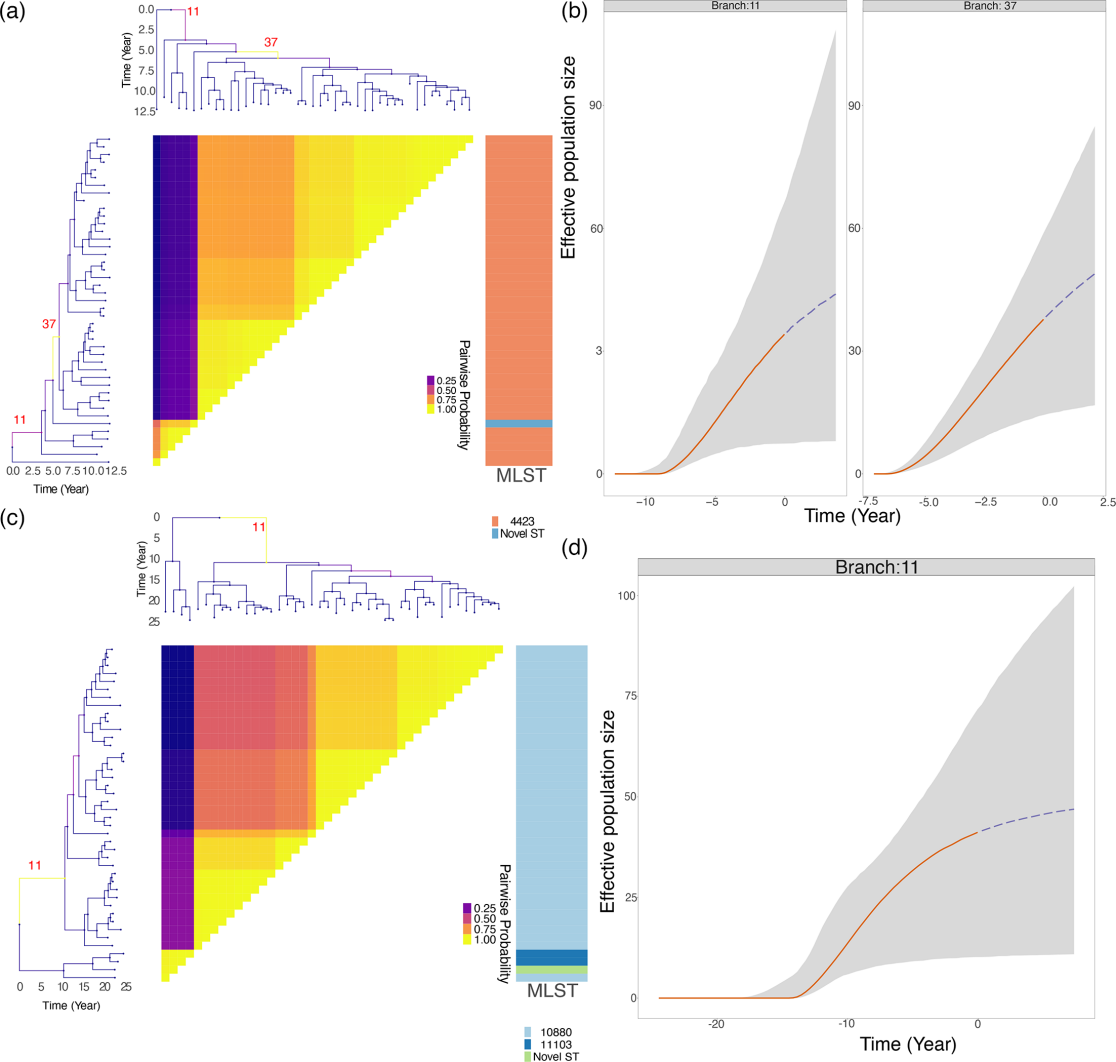
Clonal expansion and effective population of lineages showing GPSC102 lineage effects in transmission. (**a**) Dated phylogeny illustrating the expansion of GPSC102-ST4423. Pairwise matrix showing the posterior probabilities of any two genomes belonging to the same subpopulation. (**b**) Posterior summary of the inferred effective population size for GPSC102-ST4423. (**c**) Dated phylogeny illustrating the expansion of GPSC102-ST10880. Pairwise matrix showing the posterior probabilities of any two genomes belonging to the same subpopulation. (**d**) Posterior summary of the inferred effective population size for GPSC102-ST10880.

**Fig. 6.**
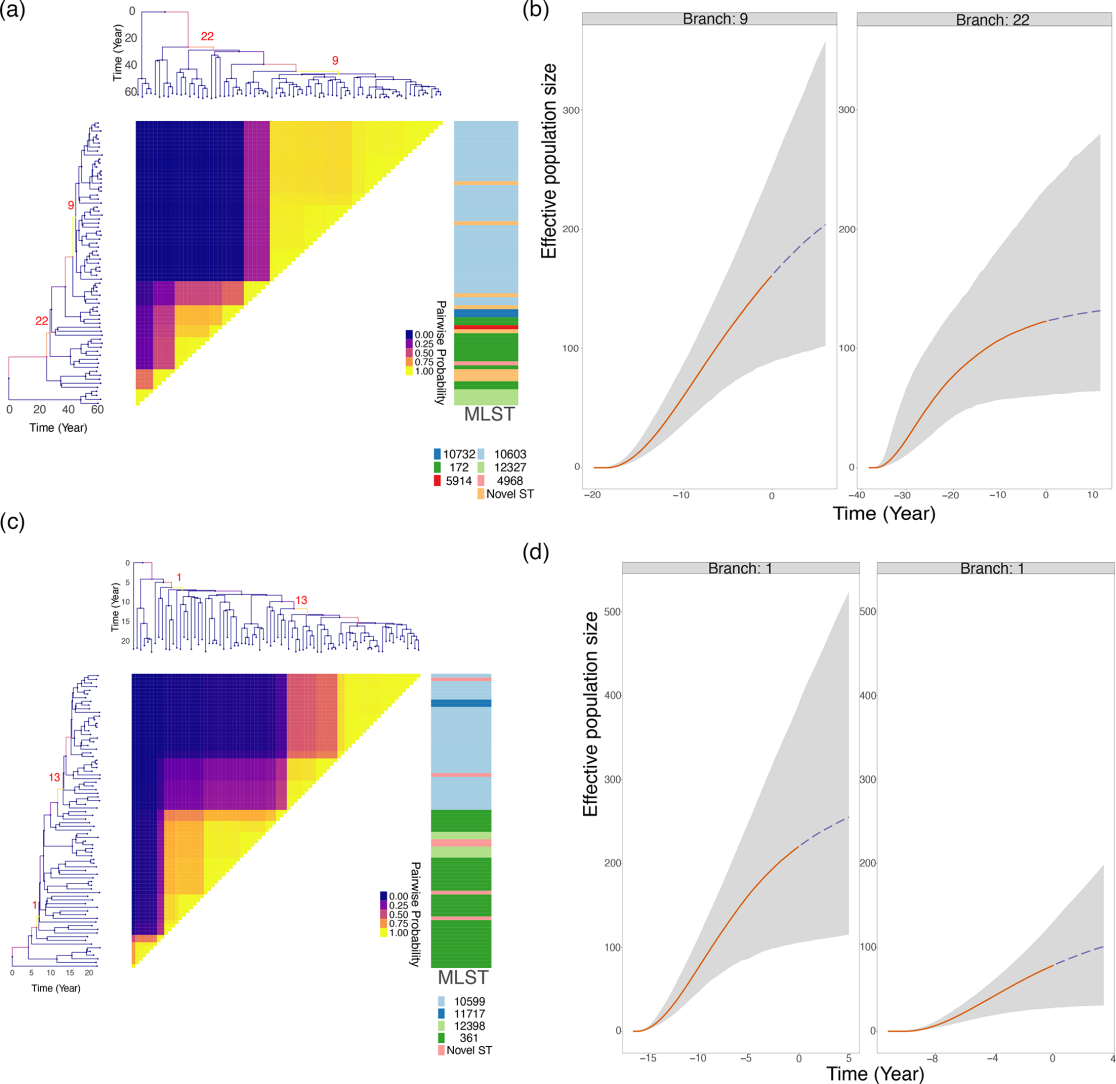
Clonal expansion and effective population of lineages showing GPSC5 lineage effects in transmission. (**a**) Dated phylogeny illustrating the expansion of GPSC5-10603. Pairwise matrix showing the posterior probabilities of any two genomes belonging to the same subpopulation. (**b**) Posterior summary of the inferred effective population size for GPSC5-10603. (**c**) Dated phylogeny illustrating the expansion of GPSC5-ST10599. Pairwise matrix showing the posterior probabilities of any two genomes belonging to the same subpopulation. (**d**) Posterior summary of the inferred effective population size for GPSC5-ST10599.

**Fig. 7.**
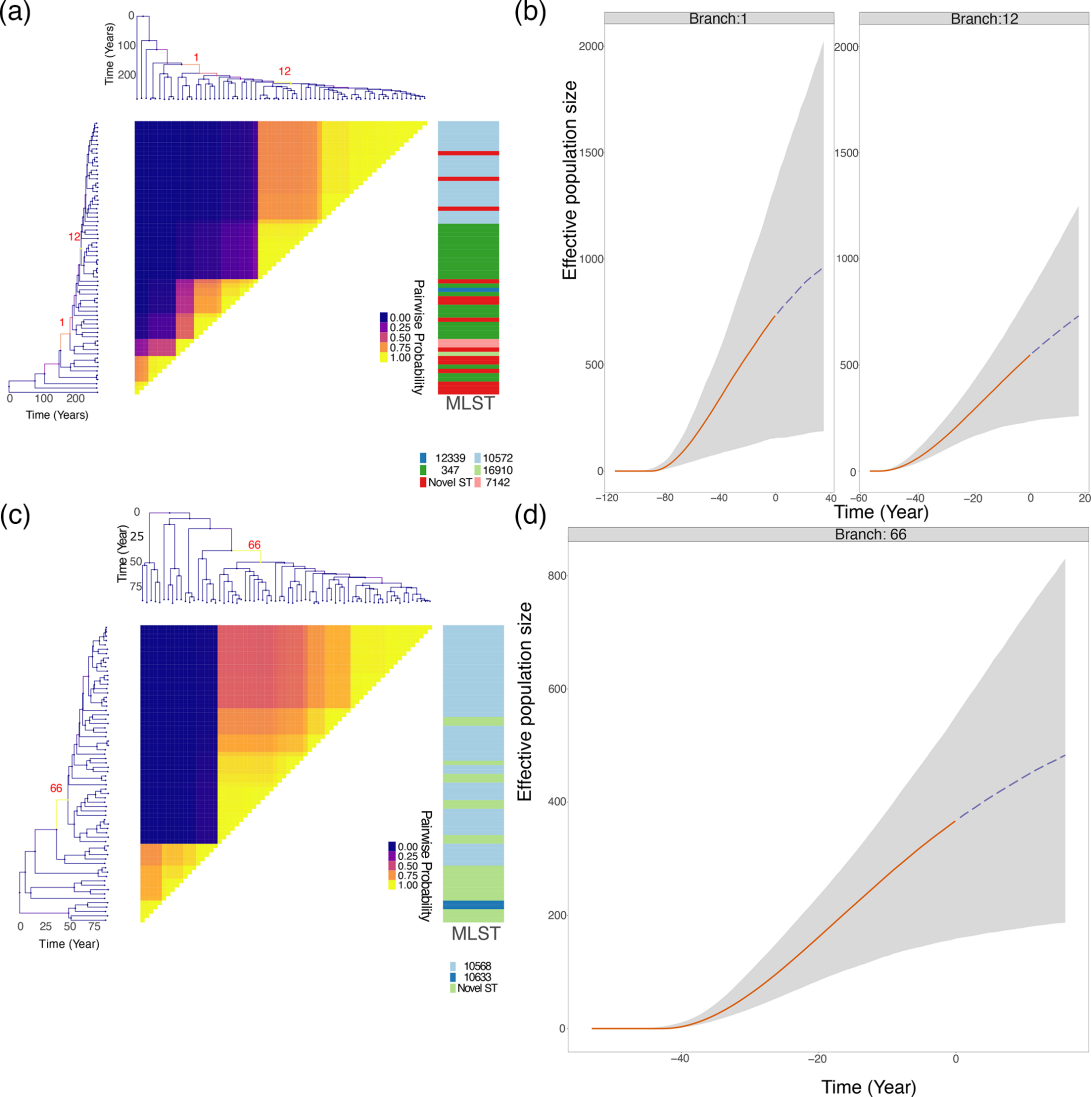
Clonal expansion and effective population size of lineages showing the effects of GPSC21 and GPSC163 in transmission. (**a**) Dated phylogeny illustrating the expansion of GPSC21. Pairwise matrix showing the posterior probabilities of any two genomes belonging to the same subpopulation. (**b**) Posterior summary of the inferred effective population size for GPSC21. (**c**) Dated phylogeny illustrating the expansion of GPSC163. Pairwise matrix showing the posterior probabilities of any two genomes belonging to the same subpopulation. (**d**) Posterior summary of the inferred effective population size for GPSC163. For the effective population size graphs, the grey area represents the 95% CI, and the lines denote the median. Solid lines indicate past effective population size inference, while dashed lines represent predictions of future effective population size. Point 0 on the x-axis corresponds to the most recent sample date, which was 201.

Finally, we reconstructed expansion timelines to assess the recency of each lineage’s growth. Within GPSC102, ST10880 (median SNP distance 23, SNP range 0–115) expanded over 10 years ago, whereas ST4423 (median SNP distance 11, SNP range 0–26) expanded within the last 5 years (Fig. 5a–d). Similarly, GPSC5-ST10599 (median SNP distance 34, SNP range 1–58) showed expansion beginning roughly 8 years ago, while ST10603’s (median SNP distance 33, SNP range 2–66) expansion dates back more than 10 years (Fig. 6a–d). In GPSC21, ST347 (median SNP distance 28, SNP range 1–73) and ST10572 (median SNP distance 55, SNP range 1–84) expanded ~60 and 40 years ago, respectively, and GPSC163-ST19568 (median SNP distance 61, range 1–96) expanded ~40 years ago (Fig. 7a–d). Taken together, these prevalence, timeline and genomic clustering data indicate that recently emergent STs with lower within-lineage SNP diversity – such as GPSC102-ST4423 and GPSC5-ST10599 – are more likely to drive current transmission than long-established lineages.

## Discussion

Using whole-genome sequencing alongside geospatial and epidemiological data, our study demonstrates that pneumococcal transmission in Blantyre is predominantly driven by pre-school children in densely populated areas. Transmission tends to be localized, occurring mainly between neighbouring households, with lineages requiring up to 4 years to become fully geographically mixed across the community. This protracted spread is largely fuelled by emergent non-vaccine serotypes exhibiting penicillin non-susceptibility. These findings corroborate previous work [15,20, 46,48], enhancing our understanding of household-to-household spread and the pace of pneumococcal dissemination in low- and middle-income urban settings. Importantly, our results show that newly emergent lineages contribute more to transmission dynamics than those that have circulated for several years, which underscores the need for targeted interventions to curb pneumococcal spread, and thereby reduce IPD. Ongoing genomic surveillance is essential to detect highly transmissible emergent lineages in such settings.

Our analysis benefited from intensive sampling – 1,618 isolates collected over 4 years in a relatively small geographic area – which affords higher resolution in tracking local transmission than comparable countrywide studies. For instance, in a study in Israel, 1,174 isolates were collected over 9 years, and in South Africa, 6,910 isolates were collected over 15 years [17]. Although previous reports describe a high force of infection among children in Malawi, our model indicates that, even here, transmission remains relatively short range, with lineages becoming fully geographically mixed within 4 years [22]. In Israel, a country of 9.6 million people at 434 people/km², lineages mixed within ~5 years [16]. By contrast, in South Africa, with 60 million inhabitants at 48 people/km², complete geographic mixing only occurred after 50 years. Such differences likely reflect a combination of socioeconomic factors, childcare practices, antimicrobial usage, immune status, transport networks, internal migration and potentially differences in circulating pneumococcal lineages or NP microbiomes [17,47, 49, 50].

Data from the pre-PCV13 era suggest that infant-to-mother and infant-to-sibling transmission chiefly drives spread within a population [51]. As expected for a respiratory pathogen, this effect was most pronounced in high-density areas of Blantyre and occurred predominantly between neighbouring households among pre-school children of similar ages. Studies in rural Kenya and The Gambia also observed a high frequency of carriage among young children, declining with age due to increased clearance rates [52,53]. Together, these observations and our localized spread patterns highlight that close-contact interactions among young children, rather than older children or adults, are the main drivers of transmission.

Bacterial factors also shape pneumococcal spread. The polysaccharide capsule, a major virulence determinant and the PCV target, can evolve through mutation and genetic exchange [54]. Animal models have shown that both capsule type and quantity influence transmission dynamics [55]. Following PCV introduction, non-vaccine serotypes with greater penicillin resistance have emerged, replacing previous vaccine serotypes [55,56]. In our study, we observed increased transmission linked to emergent, penicillin non-susceptible lineages such as GPSC5 and GPSC102, both of which have clonally expanded in multiple countries post-PCV introduction. By contrast, GPSC21, which carried the highest number of vaccine serotypes, underwent clonal expansion before vaccine rollout. These data build on our earlier observations of persistent vaccine-type carriage and demonstrate suboptimal control of such lineages. Clonal expansion of various pathogenic bacteria with enhanced transmissibility has been associated with the acquisition of AMR [56,59]. However, the driver behind the increased prevalence and transmission of penicillin non-susceptible pneumococcal isolates in our cohort remains unclear. Although beta-lactam usage has been linked to increased penicillin non-susceptibility [60], we did not collect antimicrobial consumption data. Importantly, penicillin MIC cannot be assumed to confer a universal fitness cost. Experimental studies have shown that mutations in penicillin-binding protein genes associated with increased beta-lactam resistance can impose fitness costs, but that these costs may be mitigated through compensatory mutations, often acquired via horizontal gene transfer [61]. Such compensatory evolution can restore or even enhance the fitness of resistant lineages, enabling their successful expansion in carriage and disease.

This study has several limitations affecting transmission inference. Our model cannot estimate directionality, cluster size or transmission chains in the way that network-based tools such as TransPhylo or SCOTTI can, thus affording a more granular view of spread dynamics and more accurate epidemiological parameters [62,64]. However, those tools demand detailed temporal and epidemiological data, including sampling proportions, transmission bottlenecks and within-host diversity, which were unavailable in our study. Their computational complexity also makes them impractical for larger datasets. We also lacked information on factors known to influence pneumococcal carriage, such as concurrent viral infections, pollution levels and human contact patterns [65,67], and did not include individuals without carriage, who might reveal protective host factors. Reliance on single-colony sequencing from NP swabs may have overlooked key transmission links, particularly in Malawian children who often harbour multiple pneumococcal lineages [28]. This limitation is compounded by the biological difficulty of detecting certain serotypes with very short carriage duration, a property that appears to be more strongly associated with capsular serotype than genotype [68]. Such serotypes are often highly transmissible yet transient in colonization and are typically penicillin-susceptible, meaning they may be rapidly transmitted between hosts and either cleared quickly in non-susceptible individuals or progress to disease in susceptible individuals [30]. Consequently, these lineages are likely to be systematically underrepresented in cross-sectional carriage surveys, irrespective of sequencing depth or colony selection. For example, serotype 1, a prototypical short-duration carriage serotype and a frequent cause of outbreaks, is rarely penicillin non-susceptible because its brief; consequently, it may be underrepresented when only a single colony is sequenced [15]. Employing multi-colony metagenomic sequencing could address this by detecting lower-abundance resistant strains that coexist with susceptible ones within a single host [31].

In conclusion, our analysis reveals the complexity of pneumococcal transmission dynamics in Blantyre, with both human behaviours and bacterial characteristics driving localized spread. These findings underscore the need for data-driven, targeted public health interventions that integrate epidemiological information with genomic surveillance to reduce IPD incidence. Incorporating multiple carriage sequencing data and additional epidemiological cofactors in future models would further clarify transmission patterns and inform more effective vaccine strategies aimed at interrupting transmission of disease-causing and AMR lineages, enhancing herd protection for vulnerable groups, such as young children and people living with HIV.

## Supplementary material

10.1099/mgen.0.001667 Uncited Supplementary Material 1.

10.1099/mgen.0.001667 Uncited Supplementary Material 2.

## Abbreviations

AMR: antimicrobial resistance
CI: confidence interval
GAMM: generalized additive mixed model
GLMM: generalized linear mixed-effects model
GPSC: Global Pneumococcal Sequence Cluster
IPD: invasive pneumococcal disease
MIC: minimum inhibitory concentration
OR: odds ratio
PCV: pneumococcal conjugate vaccine
PCVPA: Pneumococcal Carriage in Vulnerable Populations in Africa
R-AUC: precision–recall area under the curve
ROC-AUC: receiver operating characteristic area under the curve
RR: relative risk
SHAP: Shapley Additive exPlanations
ST: sequence type
VT: vaccine type

## References

[1] Lopez Bernal J, Panagiotopoulos N, Byers C, Garcia Vilaplana T, Boddington N (2022). Transmission dynamics of COVID-19 in household and community settings in the United Kingdom, January to March 2020. Euro Surveill.

[2] Zhou X, Ma X, Gao S, Ma Y, Gao J (2023). Measuring the worldwide spread of COVID-19 using a comprehensive modeling method. BMC Med Inform Decis Mak.

[3] Yan D, Cao H (2019). The global dynamics for an age-structured tuberculosis transmission model with the exponential progression rate. Appl Math Model.

[4] Pando C, Hazel A, Tsang LY, Razafindrina K, Andriamiadanarivo A (2023). A social network analysis model approach to understand tuberculosis transmission in remote rural Madagascar. BMC Public Health.

[5] CDC (2023). Global Pneumococcal Disease and Vaccination. https://www.cdc.gov/pneumococcal/global.html.

[6] Wahl B, O’Brien KL, Greenbaum A, Majumder A, Liu L (2018). Burden of *Streptococcus pneumoniae* and *Haemophilus* influenzae type b disease in children in the era of conjugate vaccines: global, regional, and national estimates for 2000-15. Lancet Glob Health.

[7] Rodgers GL, Whitney CG, Klugman KP (2021). Triumph of Pneumococcal conjugate vaccines: overcoming a common foe. J Infect Dis.

[8] Hoge CW, Reichler MR, Dominguez EA, Bremer JC, Mastro TD (1994). An epidemic of pneumococcal disease in an overcrowded, inadequately ventilated jail. N Engl J Med.

[9] Yahiaoui RY, den Heijer CD, van Bijnen EM, Paget WJ, Pringle M (2016). Prevalence and antibiotic resistance of commensal *Streptococcus pneumoniae* in nine European countries. Future Microbiol.

[10] Zivich PN, Grabenstein JD, Becker-Dreps SI, Weber DJ (2018). *Streptococcus pneumoniae* outbreaks and implications for transmission and control: a systematic review. Pneumonia.

[11] Gladstone RA, Lo SW, Lees JA, Croucher NJ, van Tonder AJ (2019). International genomic definition of pneumococcal lineages, to contextualise disease, antibiotic resistance and vaccine impact. EBioMedicine.

[12] WHO (2024). WHO Immunization Data portal - Detail Page. https://immunizationdata.who.int/global/wiise-detail-page.

[13] Simmons AE, Tuite AR, Buchan S, Fisman D (2024). Pneumococcal transmission dynamics during the use of a pediatric 13-valent pneumococcal conjugate vaccine in Canada. SSRN J.

[14] Horn M, Theilacker C, Sprenger R, von Eiff C, Mahar E (2023). Mathematical modeling of pneumococcal transmission dynamics in response to PCV13 infant vaccination in Germany predicts increasing IPD burden due to serotypes included in next-generation PCVs. PLoS One.

[15] Tonkin-Hill G, Ling C, Chaguza C, Salter SJ, Hinfonthong P (2022). Pneumococcal within-host diversity during colonization, transmission and treatment. Nat Microbiol.

[16] Cheng HCR, Belman S, Salje H, Dagan R, Bentley SD (2024). Estimating geographical spread of *Streptococcus pneumoniae* within Israel using genomic data. Microbial Genomics.

[17] Belman S, Lefrancq N, Nzenze S, Downs S, du Plessis M (2024). Geographical migration and fitness dynamics of *Streptococcus pneumoniae*. Nature.

[18] Swarthout TD, Henrion MYR, Thindwa D, Meiring JE, Mbewe M (2022). Waning of antibody levels induced by a 13-valent pneumococcal conjugate vaccine, using a 3 + 0 schedule, within the first year of life among children younger than 5 years in Blantyre, Malawi: an observational, population-level, serosurveillance study. Lancet Infect Dis.

[19] Kirolos A, Swarthout TD, Mataya AA, Bonomali F, Brown C (2023). Invasiveness potential of pneumococcal serotypes in children after introduction of PCV13 in Blantyre, Malawi. BMC Infect Dis.

[20] Swarthout TD, Fronterre C, Lourenço J, Obolski U, Gori A (2020). High residual carriage of vaccine-serotype *Streptococcus pneumoniae* after introduction of pneumococcal conjugate vaccine in Malawi. Nat Commun.

[21] Bar-Zeev N, Swarthout TD, Everett DB, Alaerts M, Msefula J (2021). Impact and effectiveness of 13-valent pneumococcal conjugate vaccine on population incidence of vaccine and non-vaccine serotype invasive pneumococcal disease in Blantyre, Malawi, 2006-18: prospective observational time-series and case-control studies. Lancet Glob Health.

[22] Lourenço J, Obolski U, Swarthout TD, Gori A, Bar-Zeev N (2019). Determinants of high residual post-PCV13 pneumococcal vaccine-type carriage in Blantyre, Malawi: a modelling study. BMC Med.

[23] Cave R, Kalizang’oma A, Chaguza C, Mwalukomo TS, Kamng’ona A (2024). Expansion of pneumococcal serotype 23F and 14 lineages with genotypic changes in capsule polysaccharide locus and virulence gene profiles post introduction of pneumococcal conjugate vaccine in Blantyre, Malawi. Microbial Genomics.

[24] Kalizang’oma A, Swarthout TD, Mwalukomo TS, Kamng’ona A, Brown C (2024). Clonal expansion of a *Streptococcus pneumoniae* serotype 3 capsule variant sequence type 700 with enhanced vaccine escape potential after 13-valent pneumococcal conjugate vaccine introduction. J Infect Dis.

[25] Mitchell PK, Lipsitch M, Hanage WP (2015). Carriage burden, multiple colonization and antibiotic pressure promote emergence of resistant vaccine escape pneumococci. Phil Trans R Soc B.

[26] WorldPop Open Spatial Demographic Data and Research. https://www.worldpop.org/.

[27] Obolski U, Swarthout TD, Kalizang’oma A, Mwalukomo TS, Chan JM (2023). The metabolic, virulence and antimicrobial resistance profiles of colonising *Streptococcus pneumoniae* shift after PCV13 introduction in urban Malawi. Nat Commun.

[28] Swarthout TD, Gori A, Bar-Zeev N, Kamng’ona AW, Mwalukomo TS (2020). Evaluation of pneumococcal serotyping of nasopharyngeal-carriage isolates by latex agglutination, whole-genome sequencing (PneumoCaT), and DNA microarray in a high-pneumococcal-carriage-prevalence population in Malawi. J Clin Microbiol.

[29] Lees JA, Harris SR, Tonkin-Hill G, Gladstone RA, Lo SW (2019). Fast and flexible bacterial genomic epidemiology with PopPUNK. Genome Res.

[30] Li Y, Metcalf BJ, Chochua S, Li Z, Gertz RE (2016). Penicillin-binding protein transpeptidase signatures for tracking and predicting β-lactam resistance levels in *Streptococcus pneumoniae*. mBio.

[31] Li Y, Metcalf BJ, Chochua S, Li Z, Gertz RE (2017). Validation of β-lactam minimum inhibitory concentration predictions for pneumococcal isolates with newly encountered penicillin binding protein (PBP) sequences. BMC Genomics.

[32] The European Committee on Antimicrobial Susceptibility Testing (2021). Breakpoint tables for interpretation of mics and zone diameters. http://www.eucast.org.

[33] Schwengers O, Hain T, Chakraborty T, Goesmann A (2020). ReferenceSeeker: rapid determination of appropriate reference genomes. J Open Source Softw.

[34] Benson DA, Karsch-Mizrachi I, Lipman DJ, Ostell J, Sayers EW (2011). GenBank. Nucleic Acids Res.

[35] Kille B, Nute MG, Huang V, Kim E, Phillippy AM (2024). Parsnp 2.0: scalable core-genome alignment for massive microbial datasets. Bioinformatics.

[36] Croucher NJ, Page AJ, Connor TR, Delaney AJ, Keane JA (2015). Rapid phylogenetic analysis of large samples of recombinant bacterial whole genome sequences using Gubbins. Nucleic Acids Res.

[37] Didelot X, Croucher NJ, Bentley SD, Harris SR, Wilson DJ (2018). Bayesian inference of ancestral dates on bacterial phylogenetic trees. Nucleic Acids Res.

[38] Neal EFG, Chan J, Nguyen CD, Russell FM (2022). Factors associated with pneumococcal nasopharyngeal carriage: a systematic review. PLOS Glob Public Health.

[39] Obolski U, Gori A, Lourenço J, Thompson C, Thompson R (2019). Identifying genes associated with invasive disease in *S. pneumoniae* by applying a machine learning approach to whole genome sequence typing data. Sci Rep.

[40] Lourenço J, Watkins ER, Obolski U, Peacock SJ, Morris C (2017). Lineage structure of *Streptococcus pneumoniae* may be driven by immune selection on the groEL heat-shock protein. Sci Rep.

[41] Lees JA, Galardini M, Bentley SD, Weiser JN, Corander J (2018). pyseer: a comprehensive tool for microbial pangenome-wide association studies. Bioinformatics.

[42] Earle SG, Wu C-H, Charlesworth J, Stoesser N, Gordon NC (2016). Identifying lineage effects when controlling for population structure improves power in bacterial association studies. Nat Microbiol.

[43] Ondov BD, Treangen TJ, Melsted P, Mallonee AB, Bergman NH (2016). Mash: fast genome and metagenome distance estimation using MinHash. Genome Biol.

[44] Helekal D, Ledda A, Volz E, Wyllie D, Didelot X (2022). Bayesian inference of clonal expansions in a dated phylogeny. Syst Biol.

[45] Obolski U, Lourenço J, Thompson C, Thompson R, Gori A (2018). Vaccination can drive an increase in frequencies of antibiotic resistance among nonvaccine serotypes of *Streptococcus pneumoniae*. Proc Natl Acad Sci USA.

[46] Melegaro A, Gay NJ, Medley GF (2004). Estimating the transmission parameters of pneumococcal carriage in households. Epidemiol Infect.

[47] Dunne EM, Choummanivong M, Neal EFG, Stanhope K, Nguyen CD (2019). Factors associated with pneumococcal carriage and density in infants and young children in Laos PDR. PLoS One.

[48] O’Brien KL, Wolfson LJ, Watt JP, Henkle E, Deloria-Knoll M (2009). Burden of disease caused by *Streptococcus pneumoniae* in children younger than 5 years: global estimates. The Lancet.

[49] Lapidot R, Faits T, Ismail A, Allam M, Khumalo Z (2022). Nasopharyngeal dysbiosis precedes the development of lower respiratory tract infections in young infants, a longitudinal infant cohort study. Gates Open Res.

[50] Camelo-Castillo A, Henares D, Brotons P, Galiana A, Rodríguez JC (2019). Nasopharyngeal microbiota in children with invasive pneumococcal disease: identification of bacteria with potential disease-promoting and protective effects. Front Microbiol.

[51] Heinsbroek E, Tafatatha T, Chisambo C, Phiri A, Mwiba O (2016). Pneumococcal Acquisition Among infants exposed to HIV in Rural Malawi: a longitudinal household study. Am J Epidemiol.

[52] Hill PC, Townend J, Antonio M, Akisanya B, Ebruke C (2010). Transmission of *Streptococcus pneumoniae* in rural Gambian villages: a longitudinal study. Clin Infect Dis.

[53] Lipsitch M, Abdullahi O, D’Amour A, Xie W, Weinberger DM (2012). Estimating rates of carriage acquisition and clearance and competitive ability for pneumococcal serotypes in Kenya with a Markov transition model. Epidemiology.

[54] Wyres KL, Lambertsen LM, Croucher NJ, McGee L, von Gottberg A (2013). Pneumococcal capsular switching: a historical perspective. J Infect Dis.

[55] Zafar MA, Wang Y, Hamaguchi S, Weiser JN (2017). Host-to-Host transmission of *Streptococcus pneumoniae* Is driven by its inflammatory toxin, pneumolysin. Cell Host Microbe.

[56] Steinig EJ, Duchene S, Robinson DA, Monecke S, Yokoyama M (2019). Evolution and global transmission of a multidrug-resistant, community-associated methicillin-resistant *Staphylococcus aureus* lineage from the Indian subcontinent. mBio.

[57] Miyoshi-Akiyama T, Tada T, Ohmagari N, Viet Hung N, Tharavichitkul P (2017). Emergence and spread of epidemic multidrug-resistant *Pseudomonas aeruginosa*. Genome Biol Evol.

[58] Zarrilli R, Pournaras S, Giannouli M, Tsakris A (2013). Global evolution of multidrug-resistant *Acinetobacter baumannii* clonal lineages. Int J Antimicrob Agents.

[59] Chung The H, Pham P, Ha Thanh T, Phuong LVK, Yen NP (2023). Multidrug resistance plasmids underlie clonal expansions and international spread of *Salmonella* enterica serotype 1,4,[5],12:i:- ST34 in Southeast Asia. Commun Biol.

[60] Nasrin D, Collignon PJ, Roberts L, Wilson EJ, Pilotto LS (2002). Effect of beta lactam antibiotic use in children on pneumococcal resistance to penicillin: prospective cohort study. BMJ.

[61] Albarracín Orio AG, Piñas GE, Cortes PR, Cian MB, Echenique J (2011). Compensatory evolution of pbp mutations restores the fitness cost imposed by β-lactam resistance in *Streptococcus pneumoniae*. PLoS Pathog.

[62] Klinkenberg D, Backer JA, Didelot X, Colijn C, Wallinga J (2017). Simultaneous inference of phylogenetic and transmission trees in infectious disease outbreaks. PLOS Comput Biol.

[63] De Maio N, Wu C-H, Wilson DJ (2016). SCOTTI: efficient reconstruction of transmission within outbreaks with the structured coalescent. PLOS Comput Biol.

[64] Sobkowiak B, Romanowski K, Sekirov I, Gardy JL, Johnston JC (2023). Comparing *Mycobacterium tuberculosis* transmission reconstruction models from whole genome sequence data. Epidemiol Infect.

[65] Bennett JC, Emanuels A, Heimonen J, O’Hanlon J, Hughes JP (2023). *Streptococcus pneumoniae* nasal carriage patterns with and without common respiratory virus detections in households in Seattle, WA, USA before and during the COVID-19 pandemic. Front Pediatr.

[66] Beentjes D, Shears RK, French N, Neill DR, Kadioglu A (2022). Mechanistic insights into the impact of air pollution on pneumococcal pathogenesis and transmission. Am J Respir Crit Care Med.

[67] Neal EFG, Chan J, Nguyen CD, Russell FM (2022). Factors associated with pneumococcal nasopharyngeal carriage: a systematic review. PLoS Glob Public Health.

[68] Metcalf BJ, Waldetoft KW, Beall BW, Brown SP (2023). Variation in pneumococcal invasiveness metrics is driven by serotype carriage duration and initial risk of disease. Epidemics.

