# Supplementary material for: Genomic and statistical models to characterize Streptococcus pneumoniae transmission patterns in Malawi
Source: Microb Genom. 2026 Apr 9;12(4):001667. doi: 10.1099/mgen.0.001667 (PMC13293304; doi:10.1099/mgen.0.001667)
Supplement: Uncited Supplementary Material 1. [file mgen-12-01667-s001.pdf]

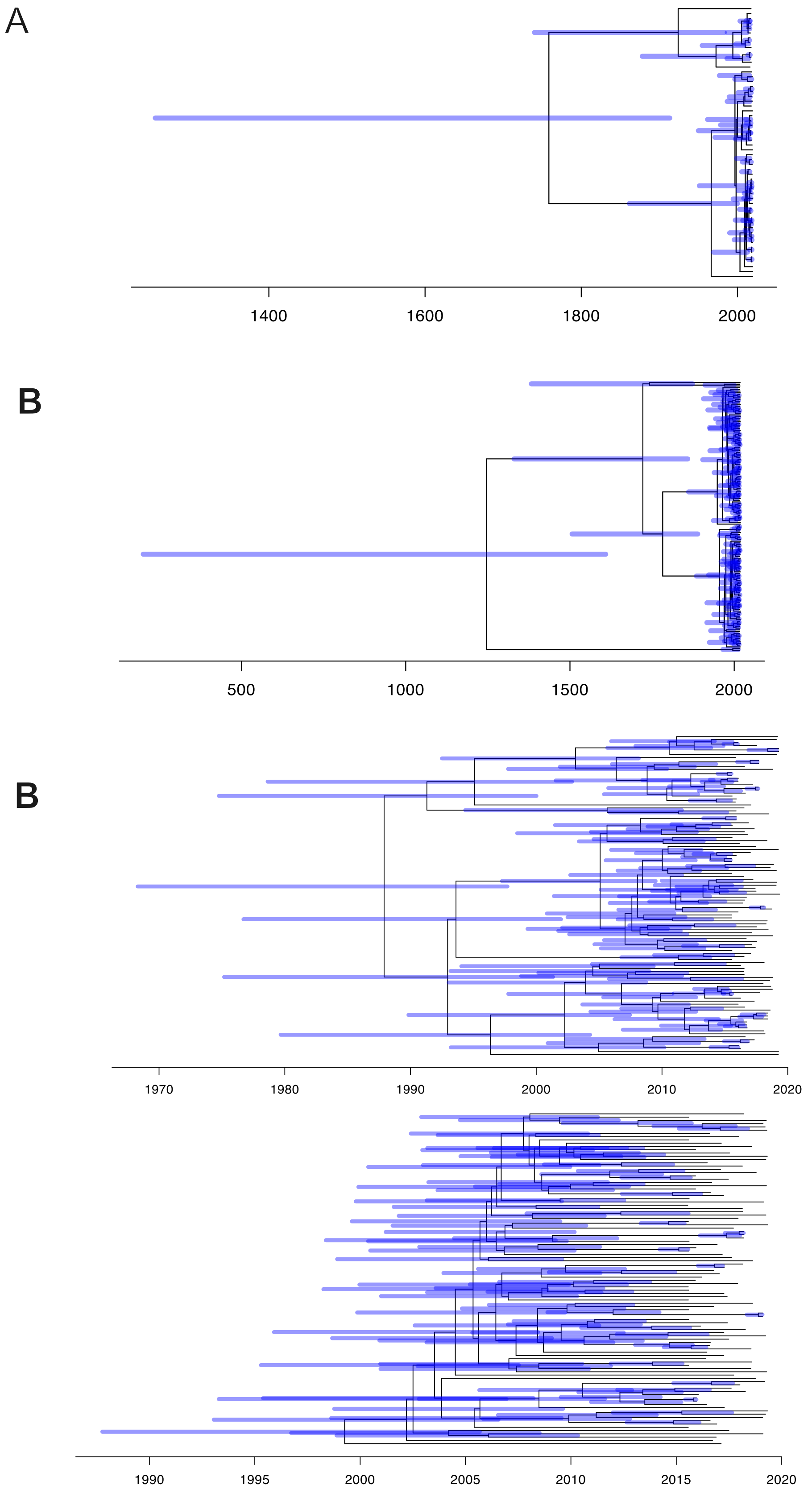

Figure S1. Example time-calibrated phylogenetic trees inferred using BactDating after reaching an effective sample size (ESS) of over 100, with 95% credible intervals (CIs) shown

in blue for: (A) GPSC102, (B) GPSC5, (C) GPSC92, and (D) GPSC65.

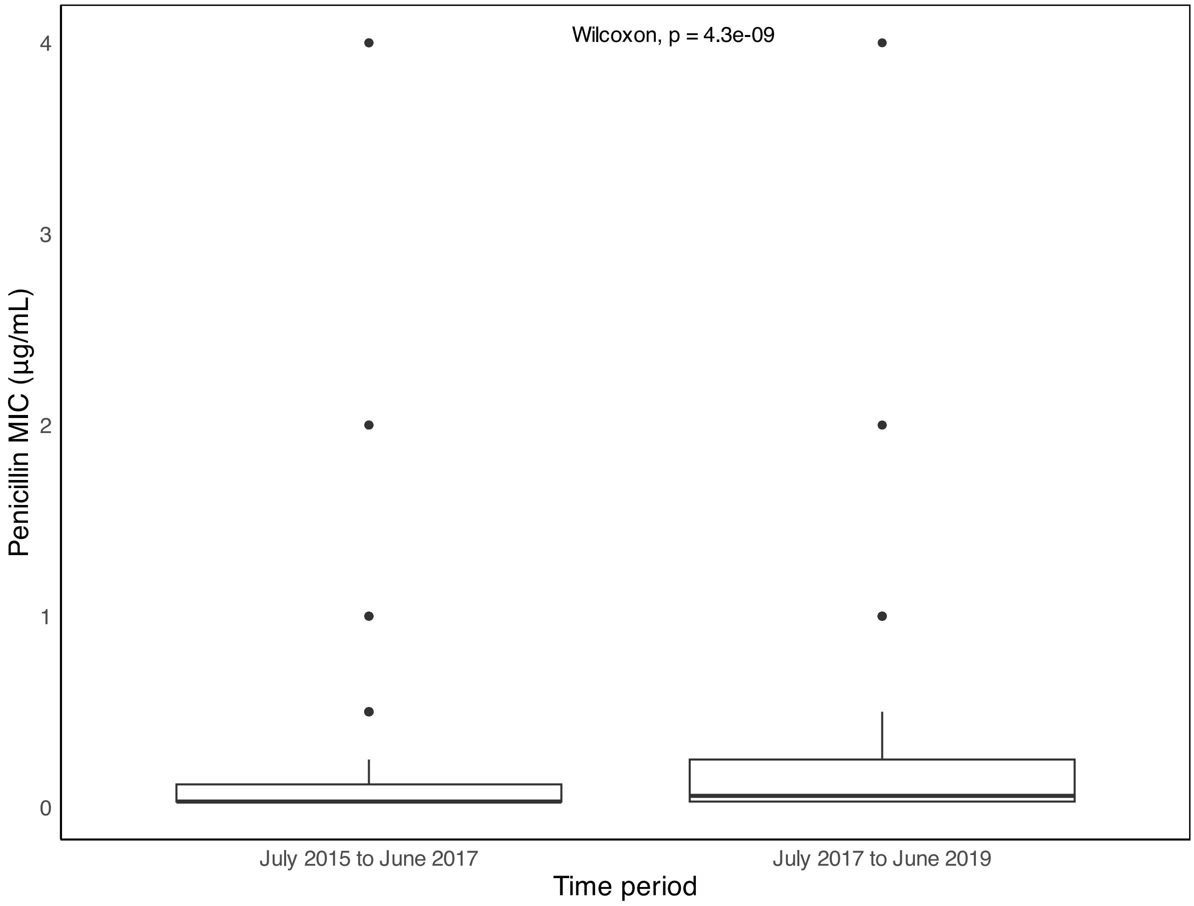

Figure S2: Boxplot showing Penicillin MIC change between early and late survey time points in the PCVPA dataset.

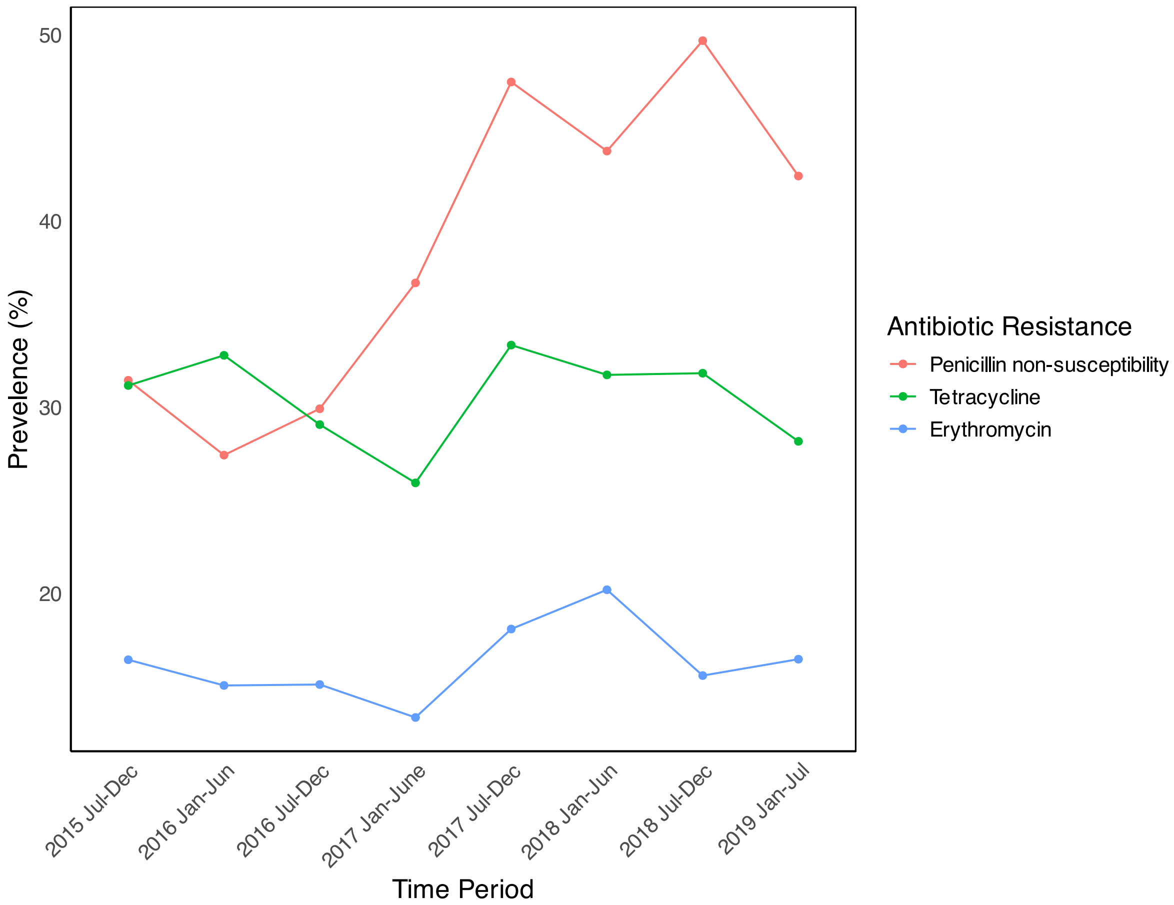

Figure S3: Line graph showing change of antibiotic resistance prevalence change during 8 separate time periods during the survey.

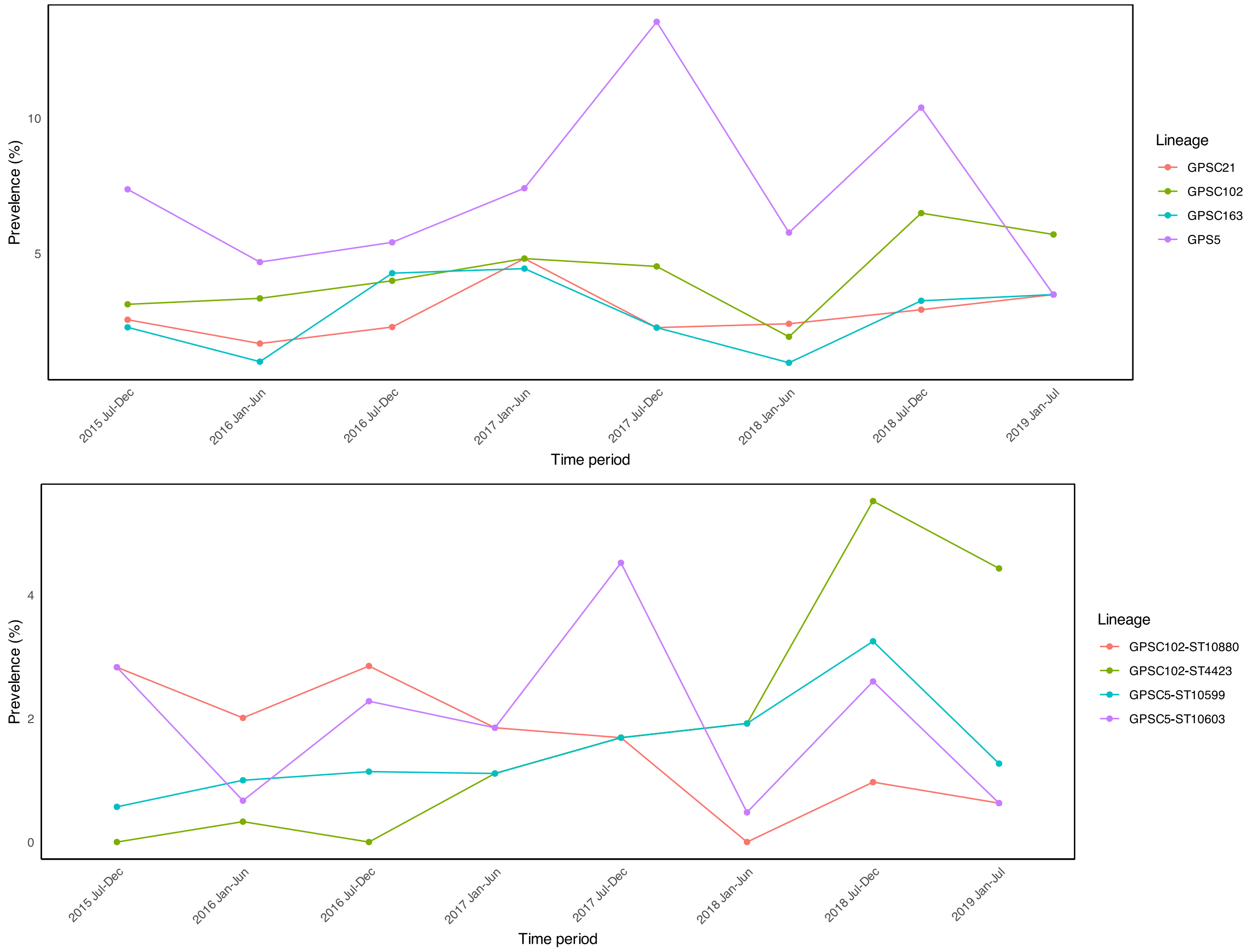

Figure S4: Line graph showing prevalence change of lineages that had significant lineage effect in transmission in the PCVPA dataset A) by their GPSC, B) By their MLST
